# Comparison of the inoculum size effects of antibiotics on IMP-6 β-lactamase-producing Enterobacteriaceae co-harboring plasmid-mediated quinolone resistance genes

**DOI:** 10.1371/journal.pone.0225210

**Published:** 2019-11-13

**Authors:** Yoshihiko Ogawa, Ryuichi Nakano, Kei Kasahara, Tomoki Mizuno, Nobuyasu Hirai, Akiyo Nakano, Yuki Suzuki, Naoki Kakuta, Takashi Masui, Hisakazu Yano, Keiichi Mikasa

**Affiliations:** 1 Center for Infectious Diseases, Nara Medical University, Kashihara, Japan; 2 Department of Microbiology and Infectious Diseases, Nara Medical University, Kashihara, Japan; Institute of Medical Sciences, Banaras Hindu University, INDIA

## Abstract

Almost all cases of carbapenemase-producing Enterobacteriaceae infections in Japan are caused by *bla*_IMP_-positive Enterobacteriaceae (especially *bla*_IMP-6_) and infections caused by other types of carbapenemase-producing Enterobacteriaceae are quite rare. We examined drug resistance genes co-harboring with *bla*_IMP-6_ and their inoculum size effects. We screened β-lactamase genes, plasmid-mediated quinolone resistance (PMQR) genes, and aminoglycoside-modifying enzyme genes by PCR and performed sequencing for 14 *bla*_IMP-6_-positive Enterobacteriaceae. Further, all PMQR-positive isolates were submitted to conjugation and inoculum effect evaluation. Our data showed that 13 of the 14 isolates harbored CTX-M-2 and one co-harbored CTX-M-2 and CTX-M-1 as extended-spectrum β-lactamases. All isolates carried one or more PMQRs; *aac(6’)-Ib-cr* was the most prevalent (92.8%), and was followed by *oqxA* (64.3%), *qnrS* (50%), *oqxAB* (21.4%), and *qnrB* (14.3%). However, *Klebsiella pneumoniae* contains chromosomal OqxAB. Inoculum size effects were significant in all strains for meropenem, 13 strains for imipenem, 7 for levofloxacin, and 3 for amikacin. We observed that 11 of the experimental strains (100%), 8 strains (72.7%), and 1 strain showed inoculum size effects for meropenem, imipenem, and amikacin, respectively. However, four strains harbored *qnr* genes and two strains harbored *qnr* genes and QRDR mutations concurrently; no inoculum size effect was seen for levofloxacin. The *bla*_IMP-6_-positive Enterobacteriaceae that we studied was found to harbor at least one plasmid-mediated drug resistance gene. The inoculum size effect for carbapenems was thought to be mainly due to IMP-6-type metallo-β-lactamase; however *qnrB* and *qnrS* also had a minimal impact on the inoculum size effect for levofloxacin.

## Introduction

Antimicrobial resistance in Gram-negative bacteria is an emerging and serious global threat to public health. Most importantly, carbapenemase-producing Enterobacteriaceae (CPE) confers broad resistance to most β-lactam antibiotics including the carbapenems [1–3]. Although the number of CPE infection cases is increasing, the optimal treatment paradigm for CPE infections has not been well defined. Furthermore, there are numerous different types of carbapenemase enzymes, such as the IMP, VIM, KPC, OXA-48-like, and NDM enzymes, each conferring varying spectrums of resistance.

Much of the existing knowledge arises from reviews of case series and retrospective studies on the VIM- or KPC-producing CPEs which are now widespread in Europe and the United States. Based on published data, combination therapy is recommended for CPE infections with reference to the results of drug susceptibility tests, even when the organisms are susceptible to carbapenems. Almost all cases of CPE infection in Japan have been caused by *bla*_IMP_-positive Gram-negative bacteria (especially *bla*_IMP-6_) and cases of infection caused by other types of CPE are quite rare [4]. Unlike other types of CPE, *bla*_IMP_-positive Enterobacteriaceae usually show susceptibility to imipenem [5]. Furthermore, in many cases, *bla*_IMP-6_-positive CPEs were susceptible to levofloxacin and amikacin [6]. Thus, based on the results of drug susceptibility tests, some infectious cases caused by IMP-type CPEs have been treated with quinolone monotherapy [7].

Quinolone resistance in Gram-negative bacteria is mostly mediated by point mutations that arise in the quinolone resistance-determining regions (QRDRs) of the gyrase and topoisomerase IV genes, leading to the modification of the target [8]. However, a previously unidentified resistance to quinolones mediated by the plasmid-mediated quinolone resistance genes (PMQRs) was recognized as a drug resistance mechanism [8–10]. These genes were first identified in 1998 and included the qnr proteins, aminoglycoside acetyltransferase, and the plasmid-mediated efflux pumps QepA and OqxAB [10]. *Klebsiella pneumoniae* contains chromosomal OqxAB. *Aac(6′)-Ib-cr* is one variant of aminoglycoside acetyltransferase which was found to reduce the activity of ciprofloxacin and aminoglycosides, including amikacin [8].

The inoculum size effect is a phenomenon whereby the measured value of the minimum inhibitory concentration (MIC) changes depending on the number of bacteria and its effect on β-lactamases, including metallo-β-lactamases (MBLs), has been described previously [11,12]. More recently, the inoculum size effect for the action of quinolone on bacteria expressing PMQR genes was described [9,13]. However, in one of these studies, it was reported that only half of the transconjugants of PMQRs (2/4 strains) showed an inoculum size effect for quinolone [9]. Furthermore, there has been no study describing the inoculum size effect for strains expressing both MBL and PMQRs.

The main aim of this study was to examine the drug resistance genes found in clinical isolates which produce IMP-6-type carbapenemase and PMQRs. In addition, we assessed the inoculum size effect on the MICs of carbapenems and quinolones.

## Materials and methods

### Bacterial isolates

From April 2015 to December 2016, sequentially collected clinical isolates of *bla*_IMP-6_-positive Enterobacteriaceae from Japan were studied. They included *Escherichia coli* (n = 2) and *Klebsiella pneumoniae* (n = 12). These isolates were non-duplicated and were obtained from 12 different hospitals around West Japan; eight isolates were from urine, four from respiratory specimens, one from bile, and one from a surgical wound.

### Detection of antimicrobial resistance genes

All isolates were confirmed *bla*_IMP-6_-positive by both PCR and DNA sequencing [14], and we performed additional PCR testing for *bla*_VIM_, *bla*_KPC_, *bla*_NDM_, and *bla*_OXA-48-like_ to assess the presence or absence of additional carbapenemase genes [15]. We also performed PCR to detect the presence of extended-spectrum β-lactamases (ESBLs; TEM, SHV, CTX-M, and OXA) [16]. The presence of plasmid-mediated quinolone resistance genes (*qnrA*, *qnrB*, *qnrC*, *qnrD*, and *qnrS*), efflux pump genes (*qepA* and *oqxAB*), and aminoglycoside acetyltransferase genes (*aac(6′)-Ib* and *aac(6′)-Ib-cr*) was also assessed by PCR [17–20].

The presence of aminoglycoside-modifying enzymes (*aph(3′)-VI* and *ant(4′′)-IIa*), which were reported to reduce susceptibility to amikacin and were detected in Enterobacteriaceae, was also assessed by PCR using specific primers [21, 22]. The QRDRs in *gyrA* and *parC* were amplified as previously described [23, 24] and were sequenced to assess any co-existing chromosomal mutations [25].

### Antimicrobial susceptibility testing

The MICs were evaluated using the agar dilution method for piperacillin, cefotaxime, cefmetazole, ceftazidime, aztreonam, cefzopran, meropenem, imipenem, levofloxacin, amikacin, and colistin at the standard inoculum size according to the Clinical and Laboratory Standards Institute (CLSI) guidelines [26]. We also evaluated the MICs of levofloxacin, amikacin, imipenem, meropenem, and colistin for strains using 10- and 100-fold of the colony forming units (CFU) in the standard inoculum. All results were interpreted according to the CLSI criteria describing *in vitro* susceptibility [27]. We defined that an inoculum size effect was significant if the MICs of the antibiotics showed a ≥ 4-fold increase at 100-fold of the inoculum size compared to the standard inoculum size. We performed these tests at least twice for each strain to confirm the accuracy of the results.

### Conjugation experiments

Conjugation experiments were performed using the broth mating technique with sodium azide resistant *E*. *coli* J53 and *E*. *coli* NR 3500 containing the gyrA mutation Ser83Phe. *E*. *coli* NR3500 was obtained from *E*. *coli* J53 using an LB agar plate containing levofloxacin (MIC; 0.125 μg/mL). Transconjugants were selected on LB agar plates containing sodium azide (100 μg/mL) and cefpodoxime (8 μg/mL). Transfer of drug resistant genes (IMP-6, CTX-M-1, CTX-M-2, *qnrB*, *qnrS*, *oqxA*, *oqxB*, and *aac(6′)-Ib-cr*) was confirmed by PCR, as described above.

## Results

### Antibiotic resistant genes

For carbapenemase genes, none of the tested isolates harbored other MBL genes besides *bla*_IMP-6_. For ESBL genes, CTX-M-2 was detected in all isolates, and one isolate co-harbored both CTX-M-2 and CTX-M-1. For quinolone and/or aminoglycoside resistant genes, 13 isolates (92.8%) harbored *aac(6′)-Ib-cr*, followed by *oqxA* (9 isolates, 64.3%), *qnrS* (7 isolates, 50.0%), *oqxAB* (3 isolates, 21.4%), and *qnrB* (2 isolates, 14.3%) (Table 1). *K*. *pneumoniae* contains chromosomal OqxAB, and neither *oqxA* nor *B* were detected in *E*. *coli* or in any of the transconjugants. None of the tested isolates harbored *qnrA*, *C*, *D*, or *qepA*. In addition, none of the isolates harbored the aminoglycoside-modifying enzyme genes *aph (3′) VI* or *ant (4′′) IIa* (Table 1).

**Table 1:** Phenotypic and genotypic characteristics of the clinical isolates of *bla*_IMP-6_-positive Enterobacteriaceae.

Isolates	Organism	Resistance genes	*gyrA* mutation	*parC* mutation	MIC (μg/mL)														
					Levofloxacin			Amikacin			Imipenem			Meropenem			Colistin		
					Standard	× 10	× 100	Standard	× 10	× 100	Standard	× 10	× 100	Standard	× 10	× 100	Standard	× 10	× 100
NR286	*E*. *coli*	IMP-6, CTX-M-2, *qnrS*, *aac(6')-Ib-cr*			0.5	0.5	**8**	16	32	32	0.125	0.25	**1**	2	8	**16**	0.5	0.5	1
NR379	*E*. *coli*	IMP-6, CTX-M-2, *qnrB*, *aac(6')-Ib-cr*	Ser83Leu	0.5	2	**8**	8	16	**32**	0.5	0.5	1	4	8	**32**	1	1	1
NR411	*K*. *pneumoniae*	IMP-6, CTX-M-2, *aac(6')-Ib-cr*, *oqxAB*	Asp87Asn	4	8	**16**	2	2	**8**	0.125	0.125	**1**	2	8	**64**	1	1	2
NR417	*K*. *pneumoniae*	IMP-6, CTX-M-2, *aac(6')-Ib*, *oqxA*	Ser83Try	Ser80Ile	1	2	**4**	4	8	8	0.125	0.25	**2**	1	8	**64**	1	1	2
NR420	*K*. *pneumoniae*	IMP-6, CTX-M-2, *qnrS*, *aac(6')-Ib-cr*, *oqxA*			0.5	1	**4**	4	8	8	0.125	0.5	**2**	2	16	**64**	0.5	2	**2**
NR449	*K*. *pneumoniae*	IMP-6, CTX-M-2, *qnrS*, *aac(6')Ib-cr*, *oqxAB*			1	1	2	2	2	2	0.125	0.25	**4**	1	8	**64**	0.5	1	**2**
NR462	*K*. *pneumoniae*	IMP-6, CTX-M-2, *aac(6')-Ib-cr*, *oqxA*	Asp87Gly		1	1	1	2	2	2	0.125	0.25	**0.5**	1	2	**32**	1	1	2
NR465	*K*. *pneumoniae*	IMP-6, CTX-M-2, *qnrS*, *aac(6')-Ib-cr*, *oqxAB*			1	2	2	2	2	4	0.063	0.125	**2**	1	16	**64**	1	1	2
NR487	*K*. *pneumoniae*	IMP-6, CTX-M-2, *qnrS*, *aac(6')-Ib-cr*, *oqxA*			1	1	**4**	2	4	4	0.25	0.5	**2**	1	16	**64**	1	1	2
NR490	*K*. *pneumoniae*	IMP-6, CTX-M-2, *aac(6')-Ib-cr*, *oqxA*	Asp87Gly		4	4	4	8	8	16	0.125	0.5	**2**	1	16	**128**	1	1	2
NR496	*K*. *pneumoniae*	IMP-6, CTX-M-2, *qnrS*, *aac(6')-Ib-cr*, *oqxA*			1	1	**4**	2	4	4	0.125	0.5	**2**	1	8	**64**	1	1	2
NR499	*K*. *pneumoniae*	IMP-6, CTX-M-1, CTX-M-2, *qnrB*, *qnrS*, *aac(6')-Ib-cr*, *oqxA*			0.5	0.5	1	2	4	**8**	0.25	0.5	**2**	2	16	**128**	1	1	2
NR512	*K*. *pneumoniae*	IMP-6, CTX-M-2, *aac(6')-Ib-cr*, *oqxA*			1	2	2	2	2	4	0.125	0.25	**1**	1	8	**64**	1	1	2
NR1647	*K*. *pneumoniae*	IMP-6, CTX-M-2, *aac(6')-Ib-cr*, *oqxA*			0.5	0.5	1	2	2	2	0.25	0.25	**2**	4	16	**64**	1	1	2

Sequencing of the PCR products derived from the QRDRs in *gyrA* and *parC* showed a substitution in the QRDR of GyrA in one *E*. *coli* isolate (NR379) and four *K*. *pneumoniae* isolates (NR411, 417, 462, 490), and in the QRDR of ParC in the *K*. *pneumoniae* isolate NR417.

### Drug susceptibility tests and inoculum size effects

The range of MICs was as follows: piperacillin (64–>256 μg/mL), cefotaxime (64–>256 μg/mL), cefmetazole (64–>256 μg/mL), ceftazidime (32–>256 μg/mL), aztreonam (1–32 μg/mL, only NR 379 showed susceptibility), cefzopran (16–256 μg/mL), meropenem (1–4 μg/mL, susceptible: 8/14 isolates), imipenem (0.063–0.5 μg/mL, susceptible: 14/14), levofloxacin (0.5–4 μg/mL, susceptible: 12/14), amikacin (2–16 μg/mL, susceptible: 14/14), and colistin (0.5–1 μg/mL, susceptible: 14/14). The inoculum size effects of the antibiotic susceptibility tests are shown in Table 1. For all the antibiotics tested, the MIC values for all the isolates at 10-fold the standard inoculum size were equal to or higher than those using the standard inoculum, and those at 100-fold the standard inoculum size were equal to or higher than those using 10-fold the standard inoculum size. Inoculum size effects were observed in all isolates for meropenem (MIC range; 1–4 to 16–128 μg/mL), 13 for imipenem (MIC range; 0.063–0.5 to 0.5–2 μg/mL), 7 for levofloxacin (MIC range; 0.5–4 to 1–16 μg/mL), and 3 for amikacin (MIC range; 2–16 to 2–32 μg/mL). Based on the CLSI breakpoint in M100-S25 at the standard inoculum size, all isolates showed susceptibility to amikacin and imipenem. Two isolates (14.3%) and six isolates (42.9%) were not susceptible to levofloxacin and meropenem, respectively. On the other hand, at 100-fold the standard inoculum size, two isolates, eight isolates, and nine isolates were not susceptible to amikacin, levofloxacin, and imipenem, respectively. All isolates were resistant to meropenem at 100-fold the standard inoculum size. For colistin, the CLSI does not define the breakpoint for Enterobacteriaceae. Only 2 of 14 isolates showed inoculum size effects based on our definition, but all isolates showed susceptibility based on the EUCAST clinical breakpoint [28].

### Conjugation experiment results

Conjugation experiments were performed successfully with 8 isolates, and 11 strains were obtained (Table 2). The recipient strain (*E*. *coli* J53, NR3500) did not show an inoculum size effect and was susceptible to all the antibiotics we tested with or without the presence of QRDR mutation. Eleven isolates (100%), eight strains (72.7%), and one strain showed inoculum size effects for meropenem, imipenem, and amikacin, respectively. In addition, based on our definition, no inoculum size effect for levofloxacin was seen in the conjugant strains. Of these strains, four harbored *qnr* genes and two harbored both *qnr* genes and a QRDR mutation concurrently. When NR379/NR3500 was compared with NR 379/J53-2, NR379/NR3500 harbored the *aac(6’)-Ib-cr* gene but did not harbor the *gyrA* mutation. There was no difference in the inoculum size effect for amikacin. Thus, our experiments showed that the inoculum size effects for carbapenems were apparent with IMP-6-type carbapenemase and that these effects were greater for meropenem than for imipenem. In addition, the *qnrS* and *qnrB* genes did not provide an apparent inoculum size effect for levofloxacin between the standard inoculum size and 100-fold the standard inoculum size.

**Table 2:** Phenotypic and genotypic characteristics of the transconjugants of *bla*_IMP-6_-producing *E*. *coli*.

Strain	Resistance genes	*gyrA* mutation	MIC (μg/mL)														
			Levofloxacin			Amikacin			Imipenem			Meropenem			Colistin		
			Standard	× 10	× 100	Standard	× 10	× 100	Standard	× 10	× 100	Standard	× 10	× 100	Standard	× 10	× 100
J53 *E*. *coli*			0.031	0.031	0.063	1	1	1	0.25	0.25	0.5	0.031	0.063	0.063	0.5	1	1
NR3500		Ser83Phe	0.5	0.5	0.5	0.5	0.5	1	0.125	0.125	0.25	0.031	0.031	0.063	0.5	0.5	1
NR286/J53	IMP-6, CTX-M-2, *aac(6′)-Ib-cr*		0.031	0.031	0.031	4	4	8	0.5	0.5	**2**	1	4	**32**	0.5	0.5	1
NR379/J53-1	IMP-6, CTX-M-2, *aac(6′)-Ib-cr*		0.031	0.031	0.063	4	4	4	0.5	0.5	1	0.5	2	**32**	0.5	0.5	1
NR379/J53-2	IMP-6, CTX-M-2, *qnrB*, *aac(6′)-Ib-cr*		0.031	0.031	0.031	16	16	16	0.5	0.5	1	0.5	1	**16**	0.5	0.5	1
NR379/NR3500	IMP-6, CTX-M-2, *qnrB*	Ser83Phe	1	1	2	8	8	8	0.125	0.125	0.25	0.25	0.5	**4**	0.5	0.5	1
NR420/J53	IMP-6, CTX-M-2, *aac(6')-Ib-cr*		0.031	0.031	0.031	1	1	2	0.25	0.5	**2**	0.5	4	**32**	1	2	2
NR449/J53	IMP-6, CTX-M-2, *aac(6′)-Ib-cr*		0.031	0.031	0.063	0.5	0.5	1	0.125	0.25	**1**	0.25	2	**32**	0.25	0.5	**2**
NR462/J53	IMP-6, CTX-M-2, *aac(6')-Ib-cr*		0.031	0.031	0.063	0.5	0.5	1	0.125	0.25	**0.5**	0.25	2	**32**	0.5	0.5	**2**
NR487/J53	IMP-6, CTX-M-2, *qnrS*, *aac(6')-Ib-cr*		0.031	0.031	0.063	1	1	2	0.5	0.5	**2**	0.5	4	**32**	1	1	2
NR487/NR3500	IMP-6, CTX-M-2, *qnrS*	Ser83Phe	0.5	0.5	0.5	0.5	1	**2**	0.125	0.25	**1**	0.25	1	**16**	0.5	0.5	1
NR499/J53	IMP-6, CTX-M-2, *aac(6′)-Ib-cr*		0.031	0.031	0.031	1	1	2	0.25	0.5	**2**	0.25	1	**32**	0.5	0.5	1
NR512/J53	IMP-6, CTX-M-2, *aac(6′)-Ib-cr*		0.031	0.031	0.031	1	1	1	0.25	0.5	**1**	0.25	4	**4**	1	1	1

## Discussion

Recently, *bla*_IMP_-positive Enterobacteriaceae has become a serious problem throughout Asian countries, including Japan [4]. However, very few studies have reported other drug resistance genes besides β-lactamases in Japan. In this study, we found that *bla*_IMP-6_-positive Enterobacteriaceae harbored multiple drug resistant genes including ESBLs and PMQRs. To the best of our knowledge, this is the first report evaluating drug resistance genes other than β-lactamases and the inoculum size effects for isolates producing IMP-6-type MBLs and PMQRs.

The resistance mechanisms for antibiotics are very complicated because organisms can harbor multiple resistant genes in their plasmids or chromosomes, and some of these genes act against multiple drug classes. In our study, we focused on IMP-6-type carbapenemase and PMQRs and the impact of the inoculum size effects of these genes. Margaritis et al. reported that half of the transconjugants of PMQRs (2/4 strains) showed inoculum size effects for levofloxacin (1-, 2-, 4-, and 16-fold: MIC of 10^7^ CFU/mL per MIC of 10^5^ CFU/mL) [9]. However, they also showed that the inoculum size effects for quinolone were significant for laboratory strains which did not harbor acquired quinolone resistance genes. Rice reported that resistance to quinolone arises as a result of a combination of various resistance mechanisms [29]. We conclude that *qnr* genes had only a small impact on the inoculum size effects for levofloxacin at 100-fold of the standard inoculum size. The change might be significant at higher concentrations than the standard inoculum size. Further studies should be performed to identify other factors, such as efflux pumps or unknown mechanisms that could explain this difference. One experimental strain (NR 379 and NR 3500), which harbored the *qnrB* gene and *gyrA* mutation, showed susceptibility to levofloxacin based on the CLSI breakpoint (M100-S25) but was resistant based on the EUCAST clinical breakpoint (ver. 8.1) at 100-fold of the inoculum size [28]. Therefore, *qnr* genes should not be completely ignored if the strain shows a decreased susceptibility to levofloxacin in the setting of a huge level of organismal infection (e.g. an abscess or bacteremia).

Presently, a combination therapeutic strategy is recommended for carbapenemase-producing Enterobacteriaceae infections [1, 2, 30]. These recommendations are based on clinical experiences with VIM- or KPC-type carbapenemase-producing Enterobacteriaceae infections where the MICs for carbapenem were high. In contrast, the reported susceptibility to meropenem and imipenem among *bla*_IMP-6_-positive *E*. *coli* was approximately 70% and 100%, respectively [4]. Pang et al. have reported that most pathogens were confirmed to produce IMP-type carbapenemases and some cases were successfully treated with quinolone monotherapy as a definitive therapy based on drug susceptibility tests [7]. However, in the present study, the isolates did not show resistance to levofloxacin, amikacin, and carbapenems based on the CLSI definition. the MICs of some isolates turned out to be high enough to be resistant to these drugs, especially to meropenem (the MIC for all isolates was ≥ 16 μg/mL), at 100-fold of the standard inoculum size. In fact, it has been reported that 55.6% of infectious cases caused by KPC-type carbapenemase-producing *K*. *pneumoniae* showed susceptibility in automated drug susceptibility tests, but failed to be treated by imipenem or meropenem [31]. Thus, it might be necessary for successful antibiotic treatment to introduce aggressive interventional procedures to reduce the quantity of the organisms which produce drug resistant genes such as IMP-6 MBLs.

## Conclusions

The *bla*_IMP-6_-positive Enterobacteriaceae we studied harbored at least one plasmid-mediated drug resistance gene, other than ESBLs and carbapenemase, at the same time. Furthermore, these isolates showed inoculum size effects for levofloxacin, amikacin, and carbapenems (especially for meropenem compared with imipenem). The inoculum size effect for carbapenems was thought to be mainly due to IMP-6-type MBLs; however *qnrB* and *qnrS* also had a small impact on the inoculum size effect for levofloxacin between the standard inoculum size and 100-fold of the standard inoculum size.
